# Rosavin: Research Advances in Extraction and Synthesis, Pharmacological Activities and Therapeutic Effects on Diseases of the Characteristic Active Ingredients of *Rhodiola rosea* L.

**DOI:** 10.3390/molecules28217412

**Published:** 2023-11-03

**Authors:** Shen Wang, Yanmin Feng, Lin Zheng, Panfeng He, Jingyi Tan, Jinhui Cai, Minhua Wu, Xiaoxia Ye

**Affiliations:** 1 School of Pharmacy, Guangdong Medical University, Zhanjiang 524023, China; wangshen@gdmu.edu.cn (S.W.); 1131043180tjy@gdmu.edu.cn (J.T.); kikoyd2023@163.com (J.C.); 2 The First Clinical Medical College, Guangdong Medical University, Zhanjiang 524023, China; 13533928525@163.com (Y.F.); jacqueline_linlin@163.com (L.Z.); 13032863621@163.com (P.H.); 3 School of Basic Medicine, Guangdong Medical University, Zhanjiang 524023, China; wugdmczp@gdmu.edu.cn

**Keywords:** *Rhodiola rosea* L., rosavin, extraction and synthesis, pharmacological activity, therapeutic effect

## Abstract

*Rhodiola rosea* L. (RRL) is a popular plant in traditional medicine, and Rosavin, a characteristic ingredient of RRL, is considered one of the most important active ingredients in it. In recent years, with deepening research on its pharmacological actions, the clinical application value and demand for Rosavin have been steadily increasing. Various routes for the extraction and all-chemical or biological synthesis of Rosavin have been gradually developed for the large-scale production and broad application of Rosavin. Pharmacological studies have demonstrated that Rosavin has a variety of biological activities, including antioxidant, lipid-lowering, analgesic, antiradiation, antitumor and immunomodulation effects. Rosavin showed significant therapeutic effects on a range of chronic diseases, including neurological, digestive, respiratory and bone-related disorders during in vitro and vivo experiments, demonstrating the great potential of Rosavin as a therapeutic drug for diseases. This paper gives a comprehensive and insightful overview of Rosavin, focusing on its extraction and synthesis, pharmacological activities, progress in disease-treatment research and formulation studies, providing a reference for the production and preparation, further clinical research and applications of Rosavin in the future.

## 1. Introduction

*Rhodiola rosea* L. (RRL), which belongs to a perennial herbaceous plant of the family *Crassulaceae*, is a kind of traditional precious herbal medicine and has been used as an adaptogen, supplement, medicine or functional food for a long time in Asia and Europe. The active ingredients and effects of RRL have attracted a lot of attention and research. Rosavin is one of the main active and characteristic components of RRL. It was first isolated from RRL root by Russian botanists and chemists, who named the compound “Rosavin” because of a rose-like aroma, and found it had antifatigue and antistress effects. In recent years, with the gradual deepening of the research on its pharmacological actions and its effects on diseases, Rosavin has demonstrated a variety of biological activities and broad prospects for clinical application.

Searches in major databases (PubMed and CNKI) for a review on Rosavin were not successful. This situation suggests that it would be of considerable value to provide a brief overview of Rosavin and its biomedical activities as well as applications. This paper systematically reviews Rosavin’s sources, pharmacological activities, therapeutic effects and mechanisms on related diseases and formulation studies based on national and international literature research.

## 2. Botanical Origin and Molecular Structure of Rosavin

RRL, commonly known as Golden Root or Arctic Root, is a perennial herb of the genus *Rhodiola* L. in the family *Crassulaceae*. There are about 90 species of *Rhodiola* L., but only about 20 of them have medicinal value, the most important of which are *R. crenulate*, *R. kirilowii*, *R. cretinii* subsp., *R. quadrifida schrenk*, *R. sacra*, etc., and RRL is the most widely used of them. RRL is mainly distributed in northwestern regions of China, Northern Europe to Russia, Mongolia, Korea and Japan [1]. The plant grows in the mountains or cliffs at an altitude of 1800–2700 m in high and cold nonpolluted zones. Because its growth environment is harsh, it has strong vitality and special adaptability. Modern pharmacological studies have revealed that RRL has a variety of biological activities, such as antioxidant [2], antifatigue [3], anti-inflammatory [4,5], antidepressant [6] and antitumor [7] effects.

RRL contains a variety of active ingredients. More than one hundred compounds have been extracted from it, including volatile substances, alcohol glycosides and cyanogenic glycosides, phenylethanol and phenylpropanol compounds, flavonoids, tannin and proanthocyanidin constituents, etc. Phenylethanol and phenylpropanol compounds are considered the representative chemical constituents of RRL [8]. The phenylethanol analog Salidroside and the phenylpropanol analog Rosavin are the main active components. The phenylpropanol analogs include Rosavin, Rosarin and Rosin, which are collectively referred to as rosavins. Other species of *Rhodiola* L. do not contain rosavins or have very low levels, and therefore rosavins are considered to be the characteristic constituents of RRL [9].

**Figure 1:**
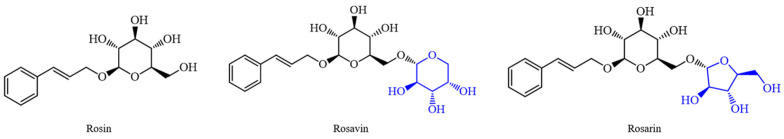
Chemical structures of the three compounds of rosavins: Rosin, Rosavin and Rosarin.

The three compounds of rosavins have similar chemical structures, among which Rosavin and Rosarin have the same relative molecular weight. Their chemical structures are both based on Rosin (Figure 1): Rosavin introduces a pyran ring substituted with a hydroxyl group at the 2,3,4 positions at the primary hydroxyl position of the pyran ring in the structure of Rosin. In the case of Rosarin, a 2,3-dihydroxyfuran structure with a hydroxymethyl substitution at the four position is introduced in the same position. In the extract of RRL, Rosavin is the most abundant component among the three compounds of rosavins, and it is also the main active ingredient of RRL. The study of Rosavin is of great significance for the development and utilization of RRL.

## 3. Extraction and Synthesis of Rosavin

### 3.1. Extraction and Isolation of Rosavin

As the main active ingredient and characteristic component of RRL, the traditional way to obtain Rosavin is to extract it from the underground rhizome of RRL. For this reason, a variety of methods have been developed for the rapid identification and detection of Rosavin in RRL extracts, such as high-performance liquid chromatography (HPLC) [10], HPLC tandem mass spectrometry (MS) [11] and HPLC using a fused-core column [12], which provide a basis for evaluating the quality of RRL extracts.

The content of Rosavin in different RRL species varies widely, and its level depends on a variety of factors, such as the plant source [13], plant harvest time [14], plant year [15], etc. Wang S. et al. used HPLC to detect the content of Rosavin in different species of *Rhodiola* L. and found that Rosavin was present in five different origins of RRL plants in China, with the content ranging from 0.08% to 0.6%. In contrast, Rosavin was absent in several sources of *R. crenulata*, *R. cretinii* subsp. and *R. kirilowii* [13].

Different extraction methods significantly affect the content of Rosavin in RRL extracts. The traditional methods mostly used water or organic extracts and combined them with auxiliary techniques such as high pressure and microwave to extract Rosavin [16,17,18]. Kosakowska O. et al. found that ethanol extraction can extract more active components from RRL than water extraction. The best results were obtained by using 70–75% ethanol, and the extracted Rosavin content reached 969.71 mg/100 g [19]. In a recent study, Tsvetov N. et al. applied natural deep eutectic solvents (NADES) for the first time to extract active ingredients from RRL. The NADES of the choline chloride and tartaric acid combination extraction were the most efficient, and the Rosavin concentration increased with time and reached its maximum of about 1000 μg/mL at 60 min of extraction [20].

The conventional isolation and purification of *Rhodiola* L. extracts require several steps, such as column chromatography, medium-pressure liquid chromatography, vacuum column chromatography, semipreparative HPLC or a combination of these techniques. Mudge E. et al. utilized high-speed countercurrent chromatography to isolate Rosavin from the methanolic extract of RRL root with a content of 3.4 mg/13.5 g and 97% purity. The method reduced losses due to irreversible adsorption and reduced solvent usage compared to conventional separation [21]. Ma C. et al. developed flash column chromatography by using ionic liquid as an extraction solvent combined with microwave extraction [22]. In the study, the separation was carried out by using a polyamide column and macroporous resin flash column in series. Finally, the 98.2% purity of Rosavin was obtained with an extraction recovery of 60.6%, providing a new method for the large-scale isolation and purification of Rosavin. To further improve the extraction efficiency of Rosavin, Yang Q. et al. used the technique of macroporous adsorption resin to separate and purify Rosavin in RRL and established a process route. Through the high physical adsorption capacity of the macroporous adsorption resin to achieve an efficient separation effect, the obtained content of Rosavin was increased from 3.00% to 68.76% with a recovery rate of 85.44%, which is helpful for the separation and purification of Rosavin in the actual industrial production [23].

### 3.2. All-Chemical and Biological Synthesis of Rosavin

**Figure 2:**
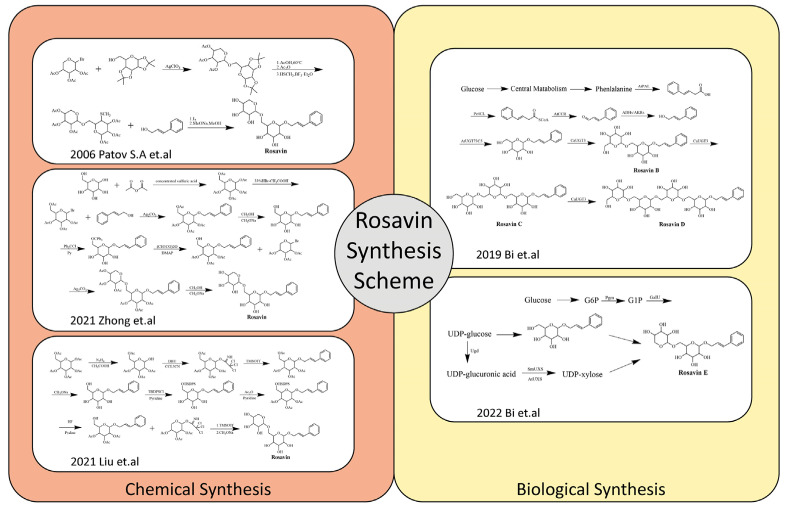
Rosavin’s chemical and biological synthesis schemes [24,25,26,27,28].

Due to the relatively single plant sources and low content of Rosavin, its extraction and isolation are complex and costly. Therefore, its current market price is rather expensive. Given this, the development of relatively simple, efficient and inexpensive chemical or biological synthesis methods for Rosavin is worth investigating. In 2006, Patov S.A. et al. developed a chemical synthesis route of Rosavin based on 1,2,3,4-diisopropylidene- D-glucopyranose and 2,3,4-tri-O-acetyl-β-L-arabinopryanosylbromide [24]. In the last two years, Chinese scholars have developed chemical synthesis methods by using safer and cheaper glucose, arabinopyranose and cinnamyl alcohol as starting materials [25,26]. In recent years, some scholars have also attempted to carry out the biosynthesis of Rosavin, and for the first time, natural Rosavin analogs were successfully obtained by using E. coli expression [27,28]. Rosavin’s chemical and biological synthesis schemes are summarized (Figure 2). Each of these approaches have their advantages and disadvantages (Table 1). The development and gradual maturation of the technology of synthesizing Rosavin by chemical or biological methods have provided the possibility of the scale-up production of Rosavin, which lays a good foundation for the further drug development of Rosavin, as well as preventive and therapeutic studies of related diseases and future clinical applications.

## 4. Pharmacological Activities of Rosavin

### 4.1. Antioxidant Effect

Oxidative stress is associated with the progression of many diseases, and the imbalance of the intracellular oxidation–reduction system leads to tissue damage, such as the aging of the organism [29]. RRL has good antioxidant activity. The four main components of RRL extract, Salidroside, Tyrosol, Rosavin and Rosarin, inhibited the activities of a superoxide anion radical (O^2−^), hydrogen peroxide (H_2_O_2_) and hypochlorous acid (HOCl) in a dose-dependent manner, with Rosavin being the most potent inhibitor of O^2−^ [30]. In a 1,1-Diphenyl-2-picrylhydrazyl (DPPH) free-radical scavenging assay and Fe^2+^ reducing-capacity assay, Rosavin also showed good antioxidant activity and a better reducing capacity than Gallic acid, Ethyl gallate and Herbacetin [31]. Hydroxyl radicals are known to be the most harmful free radicals among the reactive oxygen species in the body, capable of killing erythrocytes, degrading DNA, etc. The main active ingredients of RRL, Tyrosol, Salidroside, Rosarin and Rosavin, have a potent hydroxyl radical scavenging ability in descending order [32]. Aging is a degenerative change induced with age, which is thought to be related to the side effects of a large number of free radicals. The antiaging effect can be achieved by the effective scavenging of free radicals. In a rat model of subacute aging induced by an intraperitoneal injection of D-galactose, the Rosavin-treated group showed a dose-dependent reversal of the decline in learning and memory due to aging. It was shown that Rosavin ameliorated D-galactose-induced learning and memory decline in rats, which may be related to the rebound of the blood oxygen level and protection of enzyme activities, such as superoxide dismutase (SOD), catalase (CAT) and glutathione peroxidase (GSH-Px) and the reduction in lipid-oxidation-accumulation-products (MDA) production [33]. All of the above studies have shown that Rosavin has a strong antioxidant capacity through the effective scavenging of free radicals.

### 4.2. Lipid-Lowering Effect

The imbalance between energy intake and expenditure leads to an increase in the body’s fat content. Excessive fat accumulation in the body tends to cause a series of metabolism-related problems. Special natural components are known to reduce lipid absorption and adipogenesis, increase energy expenditure, etc. Verpeut J.L. et al. found that rats in the combined application group of RRL extract (containing 3% Rosavin and 1% Salidroside) and citrus aurantium reduced the weight of visceral fat by 30% compared with rats in the high-fat-diet model group [34]. In the lipolysis and antilipogenesis study, the experimental group containing 3% Rosavin and 1% Salidroside significantly induced higher apoptosis and lipolysis compared to the 3% Salidroside group, significantly reduced triglyceride (TG) adulteration during the maturation of preadipocytes. The expression of the genes involved in adipogenesis, SLC2A4 (solute carrier family 2, member 4) and FGF2 (fibroblast growth factor 2) was significantly decreased. However, the expression of the genes that inhibit adipogenesis, such as GATA3 (GATA binding protein 3), WNT3A (wingless-related MMTV integration site 3A) and WNT10B (wingless-related MMTV integration site 10B), was significantly increased. Mechanistic studies showed that the 3% Rosavin and 1% Salidroside group significantly downregulated the master regulators of adipogenesis, PPARG (peroxisome proliferator-activated receptor, gamma 2) and FABP4 (fatty acid binding protein 4), confirming the lipolytic and antiadipogenic activity of Rosavin [35]. A recent study demonstrated that four consecutive weeks of an intraperitoneal injection of Rosavin significantly reduced serum TG, total cholesterol (TC) and low-density lipoprotein cholesterol (LDL-C) levels and significantly increased high-density lipoprotein cholesterol (HDL-C) levels in mice fed a high-sucrose and high-fat diet [36]. The aforementioned studies have shown the potential of Rosavin in reducing lipid levels, including visceral fat weight and lipid indices, as well as regulating genes associated with adipogenesis.

### 4.3. Antifatigue Effect

Fatigue manifests as a decrease in the strength or sensitivity of cells, organs and muscles in response to stress. Adaptogen is a kind of metabolic regulator that helps adapt to the environment and enhances the body’s nonspecific resistance to harmful stimuli and injuries. RRL is a representative plant that is recognized as having plant adaptogens. Its four active components, Salidroside, Rosavin, Syringin and Triandrin, have the most potent adaptogen activity. A single dose of these adaptogens could significantly improve mental and physical performance after 30 min, lasting for at least 4–6 h [37]. In an antifatigue study in mice, Rosavin prolonged forceful swimming time, with significantly higher hepatic glycogen and myoglycogen content and a lower postexercise creatine lactate concentration [38]. Salidroside has been shown to have strong antifatigue activity [39]. Comparing Rosavin with the positive control Salidroside, the antifatigue effect of Salidroside was slightly better than that of Rosavin at the same dosage. However, there was no significant difference in the antifatigue effect between the high-concentration Rosavin group (360 mg/kg) and the Salidroside group (180 mg/kg) [38]. The above study demonstrated the antifatigue activity of Rosavin.

### 4.4. Analgesic Effect

The use of analgesics can effectively relieve the pain caused by most diseases and side effects of clinical treatments. Many active ingredients in traditional Chinese medicine have analgesic activity [40]. RRL ethanol extract (containing 2.7% Rosavin and 2.5% Salidroside) in combination with B vitamins significantly reduced formalin-injection-induced pain in mice synergistically. The analgesic effect of the RRL extract was inhibited by blocking NO synthesis, cGMP synthesis or K^+^ channels, indicating that the analgesic activity of the extract is related to the NO/cGMP/K^+^ pathway. This antinociceptive effect could be reverted in the presence of antagonists of the 5-HT_1A_, GABA/BDZs and opioid receptors, suggesting that the action mechanism of the analgesic activity of the extract involved the 5-HT_1A_ and GABA/BDZs receptors [41]. Oxaliplatin is currently the first-line treatment drug for advanced colorectal cancer, but the pain caused by an oxaliplatin injection is its typical adverse reaction. Mice developed cold pain in 3–5 days after a single oxaliplatin injection, which was significantly ameliorated by Rosavin. Moreover, the duration of its analgesic effect was positively correlated with the dosage of Rosavin. The analgesic activity of Rosavin disappeared after the 5-HT depletion by the pretreatment. Further studies found that 5-HT_1A_ receptor antagonists blocked the analgesic activity of Rosavin, confirming that Rosavin attenuates oxaliplatin-induced cold pain through the activation of 5-HT_1A_ receptors [42]. Those studies have shown that Rosavin has strong analgesic activity and the mechanism is related to 5-HT_1A_.

### 4.5. Antiradiation Effect

Radiation-protective drugs are widely used in radiation environments. But conventional antiradiation agents, such as amifostine and nilestriol, have possible drawbacks including narrow safety windows, high toxicity and poor stability [43]. It is expected that scholars will search for novel antiradiation drugs from nontoxic and low-toxic natural components. After the irradiation of AHH-1 (human peripheral blood B lymphocytes) using 10 Gy γ-rays, Isoquercitrin, Salidroside, Rosavin, Rosarin and Arbutin were all able to significantly increase the proliferative activity of AHH-1 cells after radiation damage, and Rosavin showed the strongest antiradiation activity. A total of 25 μM Rosavin increased cell viability from approximately 60% to 90% after 10 Gy γ-rays irradiation [44]. After the irradiation of IEC-6 (rat small intestinal crypt epithelial cells), the proliferation activity of the cells was enhanced to different degrees after the intervention of Salidroside, Rosavin and Arbutin, among which 12.5 μM Rosavin had the most potent protective effect, increasing the cell viability to 85.56 ± 4.93% [45]. The above studies demonstrated the strong radiation resistance of Rosavin in cells.

### 4.6. Antitumor Effect

Neovascularization in tumors is necessary for tumor growth and metastasis formation. The daily administration of 8 μg of Rosavin significantly reduced neovascularization in mice after the transplantation of L-1 sarcoma cells [46]. Recent studies have shown that Rosavin inhibits cell proliferation, clone formation, migration and invasion and promotes apoptosis and G0/G1 phase blockage. It decreases p-ERK/ERK (extracellular regulated protein kinases) and p-MEK/MEK (mitogen-activated protein kinase) protein levels, suggesting that Rosavin exerts its anti-small-cell-lung-cancer effects by inhibiting the MAPK/ERK (mitogen-activated protein kinase/extracellular regulated protein kinases) pathway [47]. These results suggest that Rosavin has potential antitumor effects and is expected to be a novel anticancer drug.

### 4.7. Immunomodulation Effect

Chen W. et al. investigated the protective effects of the main components of RRL on the immune system of mice, including Salidroside, Tyrosol and Rosavin. They found that the different active ingredients acted on different immune target cells. Rosavin has a significant proliferative effect on B lymphocytes and promotes the transformation of quiescent T lymphocytes to lymphoblasts while increasing the phagocytosis of monocytes, indicating the extensive immune activity of Rosavin [48]. It has also been shown that the main active components of RRL can alter the growth of human Jurkat T cells and apoptosis of mouse T cells, as well as the expression of surface markers and ERK phosphorylation. The strongest activity was observed for Rosavin and Rosarin, with an IC50 of 68 μM and 74 μM, respectively. Mechanistic studies have shown that Rosavin inhibits the upregulation of a TNF (tumor necrosis factor)-related apoptosis-inducing ligand (TRAIL) after T lymphocyte stimulation through the ERK pathway, whereas Rosarin shows the opposite effect [49]. The modulatory effect of Rosavin on immune cells suggests its potential in regulating autoimmune diseases.

The above pharmacological activities and detailed functional roles of Rosavin were summarized in Table 2.

## 5. The Role of Rosavin in the Treatment of Chronic Diseases and Related Mechanisms

Chronic diseases are a significant global health concern. The most common chronic diseases include cardiovascular diseases, cancer, diabetes, obesity, neurological disorders, autoimmune disorders, chronic kidney disease and many others. Chronic diseases often require long-term management and treatment, involving lifestyle modifications, slowing disease progression, preventing complications and improving life quality. The treatments often take years to obtain benefits, which may be realized more quickly by effective primary care and early intervention. This section describes Rosavin’s protective and therapeutic effects in animal model studies of neurological, digestive, respiratory and bone-related chronic diseases.

### 5.1. Nervous System Diseases

Alzheimer’s disease (AD) is a progressive neurodegenerative disease. The pathogenesis has not been fully elucidated, and no good treatment has yet been developed. Recently, some scholars conducted a study on the anti-AD efficacy of Rosavin by using a Caenorhabditis elegans dementia model. The model group nematodes produced a large amount of β-amyloid (Aβ) in vivo, while the Rosavin-treated group experienced a significantly reduced paralysis rate, prolonged nematode lifespan and improved locomotor ability. The Rosavin treatment significantly increased SOD and CAT activity in the nematodes and reduced reactive oxygen species (ROS) levels and MDA content in vivo [50]. The research suggests that Rosavin has an anti-AD effect, and the mechanism may be related to the amelioration of oxidative stress.

Microglia belong to the mononuclear phagocyte system and are intrinsic immune effector cells within the central nervous system. Microglia and their mediated neuroinflammation play an important role in the damage-repair and disease processes in the CNS and are involved in a variety of neurological disorders [51]. In a neuroinflammatory model of corticotropin-releasing hormone (CRH)-stimulated BV2 (microglial cells), RRL extract (containing 2.7% Rosavin and 1% Salidroside) reversed CRH-induced neuroinflammation by controlling NF-κB (nuclear translocation of the nuclear factor) through a mechanism that may be related to MKK2 (mitogen-activated protein kinase kinase 2), ERK1/2 and JNK (stress-activated protein kinase), leading to a reduced expression of HSP70 (heat shock protein) [52].

Depression is a common mental disorder characterized by low mood, slow thinking and delayed and reduced speech and movement as typical symptoms. Clinical studies have shown that RRL extract exhibits antidepressant activity in patients with mild to moderate depression [53]. In mouse depression modeling experiments, a single oral administration of RRL extract containing 3% Rosavin and 1% Salidroside showed significant antidepressant and anxiolytic activity, but the exact mechanism is still unclear [54].

An ischemia–reperfusion (I/R) injury is a common pathophysiological state of ischemic stroke. Recent studies have shown that different concentrations of Rosavin can inhibit the inflammatory response, neuronal apoptosis and ROS production induced by oxygen–glucose deprivation and reoxygenation (OGD/R) stimulation of human cerebral microvascular endothelial cells. In vivo studies showed that Rosavin could protect mice from brain injury in the middle cerebral artery occlusion (MCAO) model and reduce I/R-induced inflammation. At a high dose (10 mg/kg), it could almost completely inhibit neuronal apoptosis in the mouse brain and increase blood–brain barrier permeability by inhibiting autophagy. Further mechanistic studies showed that the protective effect of Rosavin on I/R mice may be related to the MAPK-mediated MMPs pathway [55].

### 5.2. Digestive System Diseases

Radiation-induced intestinal injury (RIII) is a common complication of radiation therapy for abdominal or pelvic tumors. In vitro studies have shown that the four active components in RRL, Salidroside, Herbacetin, Rosavin and Arbutin, all have protective effects on intestinal epithelial cells in the RIII model, with Rosavin having the strongest protective effect. The Rosavin therapy significantly reduced the levels of TNF-α and interleukin IL-1β; attenuated neutrophil infiltration; elevated the anti-inflammatory cytokine IL-10; moreover reduced the MDA levels; and increased the activities of the antioxidant enzymes SOD, CAT and GSH-Px. The Rosavin therapy markedly improved intestinal damage and increased the survival rate in the irradiated rats [45]. This study suggests that Rosavin can be an effective drug for the treatment of RIII. The protective mechanism is related to inhibiting the inflammatory response and oxidative stress.

Inflammatory bowel disease (IBD) is a chronic recurrent inflammatory disease of the intestinal tract, mainly including Crohn’s disease and ulcerative colitis, with a gradually increasing incidence [56]. Alterations in the balance of the intestinal microbiota are associated with the pathogenesis of IBD. *R. crenulata* extract, whose main active ingredients are Salidroside and Rosavin, significantly alleviated the pathological abnormalities of dextran sulfate sodium (DSS)-induced colitis in mice. It restored the richness and diversity of intestinal flora, increased beneficial microorganisms, reduced pathogenic bacteria and protected against colitis in mice [57]. In another study, the combination of Rosavin with 12 probiotics, prebiotics and zinc was used to attenuate DSS-induced colitis in mice. The coadministration significantly reduced the levels of proinflammatory cytokines (TNF-α, IL-6, IL-1β and IL-17) in the colonic tissues, as well as increased the levels of the Foxp3 (forkhead box P3) and the anti-inflammatory factor IL-10. Drug combinations also reduced the levels of α-SMA (α-smooth muscle actin) and Col I (collagen type I) and improved intestinal fibrosis compared to the control group. Therefore, the drug combination of Rosavin with probiotics, prebiotics and zinc can modulate inflammatory cytokines and fibrosis progression [58]. Rosavin is expected to be an important treatment drug for IBD.

Nonalcoholic fatty liver disease (NAFLD) is the most common chronic liver disease and ranges from simple steatosis to steatohepatitis (NASH), fibrosis, related liver cirrhosis and hepatocellular carcinoma [59]. In a recent study, treatment with different concentrations of Rosavin in a high-sugar–high-fat-diet-induced rat NASH model resulted in dose-dependent reductions in the serum levels of glutamic transaminase (AST), glutamic-pyruvic acid aminotransferase (ALT), alpha-fetoprotein (AFP) and total bilirubin in rats with NASH, as well as markedly reduced hepatic steatosis and hepatic fibrosis. Mechanistic studies showed that Rosavin targeted hepatic cell-death-related genes by upregulating the HSPD1, TNF, MMP-14 (matrix metalloproteinase 14), ITGB1 (integrin β1) and their upstream noncoding RNA regulators. Ultimately, Rosavin decreased the protein levels of IL-6, TNF-α and Caspase-3 (cysteine protease 3) and thereby improved the hepatic inflammation and apoptosis in NASH rats [36]. These studies have confirmed that Rosavin improves the deterioration in both liver functions and the lipid profile in NASH and has the potential to attenuate NASH disease progression.

### 5.3. Respiratory Diseases

Idiopathic pulmonary fibrosis (IPF) is a progressive and irreversible lung disease characterized by alveolar epithelial cell damage and inflammatory cell infiltration [60]. In bleomycin-induced pulmonary fibrosis mice, Rosavin significantly improved the lung index and lung histopathology structure and decreased inflammatory cell infiltration and proinflammatory cytokine expression in lung tissue, which indicated that Rosavin had a protective effect against bleomycin-induced pulmonary fibrosis in mice. Further mechanistic studies revealed that the expression of Nrf2 (nuclear factor erythroid 2-related factor 2) was increased, whereas the expression of NF-κB, TGF-β1 (transforming growth factor) and α-SMA was suppressed in the lung tissues of the Rosavin-treated mice. Additionally, Rosavin downregulated the expression of hydroxyproline (HYP) and MDA and increased the activity of SOD and GSH-Px, suggesting that the protective function of Rosavin on pulmonary fibrosis may be related to inhibiting inflammatory responses and enhancing the antioxidant capacity. The study revealed that Rosavin could be a promising drug for the treatment of pulmonary fibrosis [61].

Inflammation and oxidative stress induced by airborne fine particulate matter PM2.5 increase the morbidity and mortality of respiratory diseases. In a rat model of lung injury induced by a tracheal drip injection of PM2.5 suspension, significant ferroptosis-related ultrastructural changes were observed. An intraperitoneal injection of Rosavin alleviated the lung injury caused by PM2.5 and corrected ferroptosis-related structural alterations. Pretreatment with Rosavin reduced the levels of tissue iron and MDA and increased glutathione levels in the lung tissue. Mechanistic studies showed that Rosavin upregulated the expression of Nrf2 and other ferroptosis-related proteins, whereas a specific ferroptosis agonist RSL3 was able to reverse the protective effect of Rosavin and the intracellular phosphatidylinositol kinase (PI3K) inhibitor LY294002 decreased the upregulation of Nrf2 induced by Rosavin [62]. The study suggests that Rosavin can prevent PM2.5-induced lung injury through antiferroptosis via the PI3K/Akt/Nrf2 signaling pathway.

Sepsis is a systemic organ dysfunction caused by infection, with the lungs being the most severely infected organ. Rosavin attenuates sepsis-induced lung injuries caused by cecal ligation and puncture (CLP) in mice, inhibits the inflammatory response and reduces neutrophil extracellular trap (NET) levels and myeloperoxidase activity in CLP model mice. Mechanistic studies have shown that Rosavin inhibits the NET formation to attenuate sepsis-induced lung injury by inhibiting the MAPK/ERK/p38/JNK signaling pathway [63]. This study showed that Rosavin can be an effective drug for the treatment of sepsis.

### 5.4. Bone Diseases

Osteoarthritis (OA) is a degenerative disease that can cause pain, joint inflammation and destruction of the articular cartilage matrix, finally leading to loss of mobility. It is the main cause of disability in the elderly [64]. Cartilage degeneration is one of the most important causes of pain in OA. In a study of a monosodium iodoacetate (MIA)-induced OA rat model, the combination of Rosavin with a probiotic complex and zinc increased the femur volume and attenuated cartilage destruction in OA model rats, showing a significant anti-cartilage-degeneration effect. It also exerted an antinociceptive function by upregulating the paw-withdrawal latency (PWL), paw-withdrawal threshold (PWT) and weight-bearing capacity. The combination reduced inflammation-induced pain and joint destruction by downregulating the proinflammatory cytokine levels of IL-6 and TNF-α but enhancing IL-10 expression. The combination exerted chondroprotective effects by decreasing MMP3 and increasing the expression of tissue inhibitors of metalloproteinase TIMP3 [65]. The research revealed the cartilage-degeneration improvement and anti-inflammatory effects of the complex among MIA-induced OA rats, suggesting that Rosavin may be a candidate for OA treatment.

Bone metabolic homeostasis is maintained by osteoblast-associated bone formation and osteoclast-associated bone resorption. Excessive osteoclastogenesis or reduced osteogenesis, resulting in the dysregulation of bone homeostasis, can lead to various diseases, such as postmenopausal osteoporosis [66]. In a study of the effect of Rosavin on osteoclastogenesis, in vitro experiments showed that Rosavin inhibited osteoclastogenesis and suppressed the function of osteoclasts. It could reduce the expression of genes related to osteoclast differentiation, inhibit the phosphorylation of p65 and the inhibitory subunit of NF-κB (IκBα) induced by the osteoclast differentiation factor RANKL as well as suppress p65 nuclear translocation. Rosavin also inhibited the phosphorylation of ERK, p38 and JNK. In vivo experiments showed that compared with the control group mice, the Rosavin treatment significantly increased the number and area of bone trabeculae, bone volume and bone density and attenuated bone loss in the distal femur, suggesting that Rosavin was able to alleviate ovariectomy-induced osteoporosis in mice [67]. These studies indicated that Rosavin suppressed RANKL-induced osteoclastogenesis by blocking the NF-κB and MAPK pathways, and it may be a potential drug for the clinical treatment of osteoclastogenesis-related disorders.

**Figure 3:**
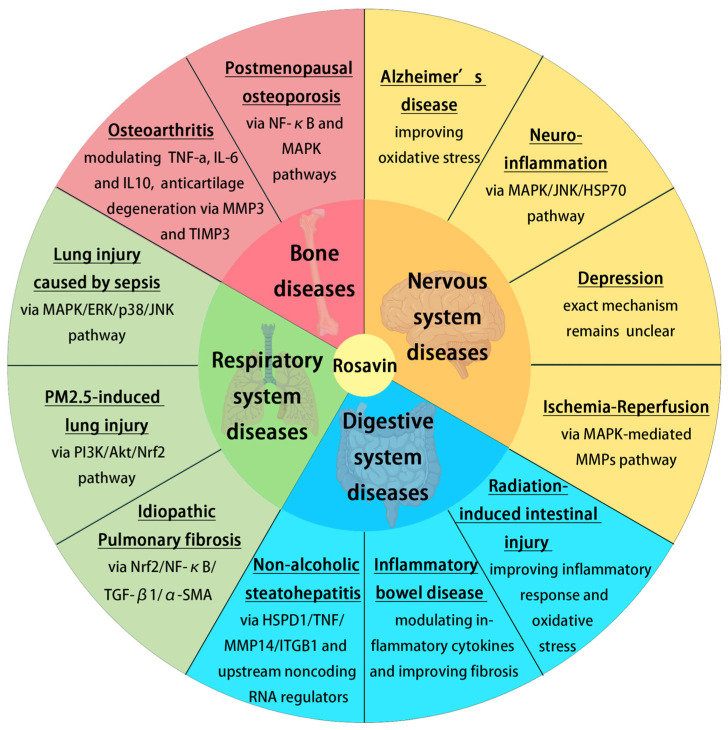
The role of Rosavin in disease treatment and its associated mechanisms (created with BioRender.com).

The previous research mentioned above established that Rosavin could be effective in in vitro and vivo models of chronic diseases, including AD, neuroinflammation, depression, I/R, RIII, IBD, NASH, IPF, PM2.5-induced lung injury, sepsis and postmenopausal osteoporosis (Figure 3). The therapeutic effect of Rosavin is associated with its anti-inflammatory and antioxidant activity, modulation of the MAPK/NF-κB pathway, etc. Rosavin may also be effective for other chronic diseases such as Parkinson’s disease, alcoholic fatty liver disease and coronary artery disease. However, there are no relevant studies on the above diseases. A lot of research is still needed on the use of Rosavin in such chronic diseases.

## 6. Drug Metabolism and Formulation Studies of Rosavin

Rosavin is a hydrophilic compound with high water solubility and a low oil–water partition coefficient. Currently, researchers mostly use ultraperformance liquid chromatography (UPLC) [68] and UPLC-MS/MS methods [69] for pharmacokinetic assays of Rosavin. The results of the plasma assay after a single administration of Rosavin to rats showed that the blood concentration of Rosavin declined rapidly after an intravenous administration of 10 mg/kg to rats with a half-life of 5.5 ± 1.3 h, whereas the concentration of Rosavin in the plasma increased gradually after a gavage administration of 20 mg/kg to rats and reached a maximum of 324.3 ± 66.9 ng/mL with a half-life of 11.6 ± 2.7 h. Compared with intravenous administration, gavage administration has a longer drug-elimination time. But the oral bioavailability (F) of Rosavin was only 2.5 ± 0.2%, which may be related to the malabsorption caused by the hydrolysis of Rosavin to glycosidic elements in the acidic environment of the digestive tract and the first-pass metabolism of the drug [68,69].

In the current studies, the gavage dosage of Rosavin is approximately 50–200 mg/kg [45,62], with a maximum dosage of 360 mg/kg [38]; most intraperitoneal doses are within 10 mg/kg [56,68], with a maximum dosage of 30 mg/kg [36]. However, there are no studies to identify Rosavin’s toxicity, and the systemic toxicological analysis in the liver and kidney after using Rosavin is still unclear.

To improve the oral effect of Rosavin, a research team improved the fast-disintegrating oral tablets of the compound Rosavin based on the traditional dosage form, using Rosavin as the main ingredient with excipients such as an ice-tablet β-cyclodextrin inclusion, lactose, microcrystalline cellulose, low-substituted hydroxypropylcellulose and crosslinked povidone. The compound tablets achieved rapid disintegration and a rapid onset of action under no water or a small amount of water [70]. In a further study, Rosavin tablets were prepared by using Rosavin as the main drug plus hydroxypropyl methylcellulose, maltol sugar powder, microcrystalline cellulose and magnesium stearate and were applied to the prevention and treatment of coronary heart disease, angina pectoris, as well as in combination with anticancer drugs to reduce the toxicity of anticancer drugs, etc. [71]. Pharmacodynamic studies showed that the Rosavin tablets could significantly prolong the weight-loaded swimming time and survival time under normobaric hypoxia in mice and improve the ability of mice to withstand high and low temperatures. The mice in the high-dose group (0.8 g/kg) experienced a significant inhibition of the tumor-growth ratio of the H22 hepatocellular carcinoma transplantation tumor. Rosavin tablets could enhance the phagocytosis function of the reticuloendothelial system and increase the percentage of peripheral blood T-lymphocytes in the H22 tumor-bearing mice, showing the effects of the Rosavin tablets in terms of their antifatigue, antistress and antitumor effects and the enhancement of immune function [71].

## 7. Summary and Outlook

As a unique component of RRL, Rosavin is a crucial active ingredient in the *Rhodiola* L. plant. Recent studies have highlighted Rosavin’s powerful multibioactivities, including its antioxidant, lipid-lowering, antifatigue, analgesic, antiradiation, antitumor and immunomodulatory effects. However, its mechanism of action has not been fully elucidated, which has encouraged more interest in further research. Existing animal model experiments have confirmed that Rosavin has significant therapeutic effects on a range of diseases such as Alzheimer’s disease, radioactive intestinal injuries, pulmonary fibrosis and osteoarthritis. These investigations show the great potential of Rosavin as a therapeutic drug for diseases and provide some basis for the future clinical treatment of related diseases.

With the deepening of the research and the emergence of broad application prospects, the demand for Rosavin is expanding. Traditional plant extraction and isolation are no longer sufficient for research and application needs. So, all-chemical and biological synthesis pathways of Rosavin have been gradually developed, which has provided the possibility of scaling up the industrial production of Rosavin.

Rosavin’s investigations have shown promising effects, but several shortcomings need to be addressed. Some of the research directions of interest are as follows. Firstly, biosynthesis should be improved to achieve large-scale production. Industrial synthetic production will be the primary source of Rosavin in the future, and biosynthesis is more favorable due to the toxicity of chemical synthesis. However, the current biosynthesis yields Rosavin analogs. Further research must clarify the key enzyme in it and improve the synthetic route to obtain Rosavin. Future research is expected to achieve a green, nontoxic and high-yield total biosynthesis, which will greatly reduce the price of Rosavin and promote its pharmaceutical formulations and research related to disease treatments. Secondly, the preparation and formulations of Rosavin are still relatively scarce. The current oral bioavailability of Rosavin is only 4.7% [70], which may be related to its poor small intestinal membrane permeability and first-pass metabolism. How to formulate Rosavin so that it can be absorbed and utilized as much as possible is an issue that needs to be investigated by pharmacy scholars, such as preparing it into phospholipid complexes, using liposomes or changing its delivery system. Thirdly, toxicological studies on Rosavin are needed. As of today, related toxicological studies on the liver and kidney after the use of Rosavin are still unclear. Finally, clinical trials are lacking. Most of the studies of Rosavin’s therapeutic effects on diseases are still at the stage of basic research. Some large-scale, well-controlled clinical trials are needed to establish its safety and efficacy.

In summary, future research will focus on the total biosynthesis, preparation research, precise mechanisms of action and the optimal use of Rosavin in various disease therapies and large-scale clinical trials. It is a broad development space worth exploring.

## Figures and Tables

**Table 1:** All-chemical or biological synthesis of Rosavin.

Typology	Raw Materials	Advantages and Disadvantages	Year and Reference
Chemical	1,2,3,4-diisopropylidene-D-glucopyranose, 2,3,4-tri-O-Acetyl-β-L- arabinopryanosylbromide	Synthesis of Rosavin through 4 steps, short synthesis route, but complex raw materials preparation and harsh catalytic reaction conditions.	2006 [24]
Chemical	Cinnamyl alcohol, fully acetyl-α- D-bromoglucose	Synthesis of Rosavin through 7 steps with an overall yield of 15.92%, low cost, mild conditions, simple operation and low contamination.	2021 [25]
Chemical	β-D-Pentaacetylglucose, arabinopyranose	Synthesis of Rosavin through 9 steps, long synthesis route; low production cost, cheap raw materials, high safety, low pollution, high yield.	2021 [26]
Biological	Glucose, *E. coli* (DH5α, BL21), gene deletion strains BPHE and BTAL	This route synthesizes a Rosavin analog, inexpensive and sustainable, but the key enzyme that catalyzes the conversion of Rosin to Rosavin is not yet known.	2019 [27]
Biological	Glucose, *E. coli* (DH5α, BL21), phenylalanine high-producing strain BPHE	This route synthesizes a Rosavin analog, green and sustainable production, batch fermentation potency of 782.0 mg/L in a 5 L bioreactor.	2022 [28]

**Table 2:** Various pharmacological activities of Rosavin.

Pharmacological Activity	Functional Roles	Experiment Models	Author and Reference
Antioxidant	inhibit the activities of O^2−^, H_2_O_2_ and HOCl	In vitro	Huang S. [30]
	inhibit DPPH activity and reduce Fe^2+^	In vitro	Zhong L. [31]
	scavenge hydroxyl radicals	In vitro	Ma T. [32]
	rebound SOD, CAT, GSH-PX enzyme activities and reduce MDA level	Mouse	Tan H. [33]
Lipid lowering	reduce the weight of visceral fat	Mouse	Verpeut J. [34]
	lipolysis and antilipogenesis through PPARG and FABP4	Primary human visceral adipocytes	Pomari E. [35]
	reduce serum TG, TC and LDL-C, increase HDL-C	Mouse	Albadawy R. [36]
Antifatigue	improve mental and physical performance	Rat	Panossian A. [37]
	prolong forceful swimming time, increase hepatic glycogen and myoglycogen content and reduce postexercise creatine lactate concentration	Mouse	Zhang H. [38]
Analgesic	reduce formalin-injection-induced pain via NO/cGMP/K+ pathway and 5-HT_1A_ receptors	Mouse	Montiel-Ruiz R. [41]
	ameliorate cold pain caused by oxaliplatin injection via 5-HT_1A_-receptors-related pathway	Mouse	Li D. [42]
Antiradiation	increase cellular proliferative activity after radiation damage	Human peripheral blood B lymphocyte line AHH-1	Ma T. [44]
	increase cellular proliferative activity after radiation damage	Rat small intestinal crypt epithelial cell line IEC-6	Zhou W. [45]
Antitumor	reduce tumor neovascularization	Mouse	Skopiñska-Ró E. [46]
	inhibit cell proliferation, migration and invasion and promote apoptosis and G0/G1 phase blockage via MAPK/ERK pathway	Human SCLC cell lines H69, H446 and H526	Liu R. [47]
Immunomodulation	enhance immune response, stimulate the proliferation of B lymphocytes, promote the transformation of T lymphocytes	Primary mouse immune cells	Chen W. [48]
	alter the growth and the apoptosis of T cells via ERK pathway	Human Jurkat cell line (ATCC TIB-152), primary mouse T cells	Marchev A. [49]

## Data Availability

Not applicable.
